# The Water Health Open Knowledge Graph

**DOI:** 10.1038/s41597-025-04537-4

**Published:** 2025-02-15

**Authors:** Anna Sofia Lippolis, Giorgia Lodi, Andrea Giovanni Nuzzolese

**Affiliations:** 1 Institute of Cognitive Sciences and Technologies, CNR, Bologna and Rome, Italy; 2 University of Bologna, Bologna, Italy

**Keywords:** Water resources, Hydrology

## Abstract

Global sustainability challenges have recently led to an increasing interest in the management of water and health resources. Thus, the availability of effective, meaningful and open data is crucial to address those issues in the broader context of the Sustainable Development Goals of clean water and sanitation as targeted by the United Nations. In this paper, we present the Water Health Open Knowledge Graph (WHOW-KG) along with its design methodology and analysis on impact. Developed in the context of the EU-funded WHOW (Water Health Open Knowledge) project, the WHOW-KG is a semantic knowledge graph that models data on water consumption, pollution, extreme weather events, infectious disease rates and drug distribution. Indeed, it aims at supporting a wide range of applications: from knowledge discovery to decision-making, making it a valuable resource for researchers, policymakers, and practitioners in the water and health domains. The WHOW-KG consists of a network of five ontologies and related linked open data, modelled according to those ontologies. As a fully distributed system, it is sustainable over time, can handle large datasets, and allows data providers full control, establishing it as a vital European asset in the fields of water consumption and pollution.

## Introduction

Interest in water and sanitation management has grown in recent years driven by global sustainability challenges that prioritise, among the others, clean water and sanitation, as outlined in the UN Sustainable Development Goals^1^.

To provide effective responses to these global issues, the availability of high quality and open data becomes an essential requirement. However, the heterogeneity and complexity of water and health data, when available, can pose significant challenges. Not only data is heterogeneous both in format and in semantics, but mostly it does not guarantee, at any level, the FAIR principles^2^, designed to assess to what extent data is Findable, Accessible, Interoperable, and Reusable. FAIR principles aim at enhancing data sharing and reuse in both human and machine contexts. More specifically, findable refers to the fact that both data and metadata should be easy to locate for both humans and machines. This includes assigning persistent identifiers (e.g. DOIs) and ensuring metadata is richly described and indexed in searchable repositories. Accessible is about the use of standardised protocols to retrievable data and metadata. Instead, interoperable refers to the use of standardised formats, vocabularies, ontologies, and frameworks to ensure compatibility with other datasets, tools, and workflows, facilitating integration across disciplines. Finally, reusable refers to the specification of rich and detailed metadata to describe data, by including clear licensing terms, and adhere reproducible processes hence supporting reuse by third parties. However, some research studies^3–5^ show that (open) data are often not findable, and not accessible nor interoperable. This claim is especially relevant since the FAIR principles currently do not include detailed guidelines on data or software quality, nor do they address issues of trustworthiness or content interoperability—gaps that ontologies can help bridge^6^. Furthermore, the absence of clear licensing frameworks makes it common to encounter datasets with unspecified licenses, rendering direct reuse of the data impossible^7^. In response, only a few ontological modelling works have emerged to represent this fragmented knowledge within a FAIR framework, aiming to cater to the need for coverage of heterogeneous datasets in the international landscape.

This paper introduces the Water Health Open Knowledge Graph (WHOW-KG), which is the first European open distributed knowledge graph aimed at linking, using a common semantics, data on water consumption and quality with health parameters (e.g., infectious diseases rates, general health conditions of the population). Designed to understand the impact of water-related climate events, water quality, and water consumption on health, it provides a harmonised data layer that can be re-used for analysis, research, and development of innovative services and applications. The project’s primary driver was to establish a sustainable methodology for open knowledge graph production to ensure authoritativeness, timeliness, semantic accuracy, and consistency data quality characteristics, as well as metadata compliance with the European DCAT-AP profile^8^ and related national and thematic extensions.

The WHOW-KG currently consists of more than 100 millions of RDF triples from 19 selected datasets according to three use cases. The WHOW-KG is distributed and it is available via three SPARQL endpoints: two endpoints available from two data providers, i.e. Lombardy Region (https://lod.dati.lombardia.it/sparql) and ISPRA - Italian National Institute of Environmental Research (https://dati.isprambiente.it/sparql), and one endpoint from CNR - Institute of Cognitive Sciences and Technologies (https://semscout.istc.cnr.it/sparql). The Lombardy region was included in this project as one of the consortium partners (i.e. ARIA SpA) is the in-house company of the Lombardy region responsible for creating, managing, and curating open data on behalf of the region. Furthermore, Lombardy is recognised for its excellence in open data production. Those open data include extensive datasets covering microbiological, chemical, and physical parameters of water. Additional data from the Region’s Agency for Environmental Protection (ARPA) and its epidemiological observatory contribute to a comprehensive overview of the topics covered by WHOW, from bathing water quality to infectious diseases and associated health services. All the resources from the Lombardy Region are licensed under the Creative Commons Public Domain License (CC0) and the ones from ISPRA under the Creative Commons Attribution 4.0 International (CC-BY 4.0) License.

In summary, this paper presents the following contributions:

- An analysis of the **five WHOW ontologies**: the Hydrography ontology, the Water Monitoring ontology, the Water Indicator Ontology, the Weather Monitoring ontology, and the Health Monitoring ontology; including a review of the state of the art in terms of similar works in both domains of water and health;
- The **WHOW-KG** and a discussion of its impact;
- A **design methodology** to support data providers in the publication of FAIR, highly extensible and sustainable Linked Open Data.

## Related Work

Knowledge graphs have increasingly become a fundamental tool in enhancing the representation and analysis of monitoring data across various domains. Thanks to ontologies—explicit specifications of a conceptualisation for a given domain of interest^9^—knowledge graphs enable interoperability and knowledge sharing by design, thus fostering the easy adoption of best practices. This is particularly evident in the context of environmental monitoring, where technologies like the Semantic Sensor Network Ontology (SSN Ontology)^10^ are crucial for representing sensors and observational processes. Furthermore, the SSN Ontology implements, for the majority of its semantic elements, the ISO 19156 Observations and Measurements (O&M) standard^11^, used also as a reference model in the INSPIRE geoportal^12^. The most prominent environmental ontology is ENVO^13^, a general-purpose ontology about different kinds of environmental entities. Originally developed as a controlled vocabulary to support the metadata checklists of the Genomic Standards Consortium, it differs from the WHOW ontology network in its lack of adherence to the specific use cases of water and health monitoring, as well as in its wide range purpose^1^.

^1^For a terminological comparison between the WHOW ontology network and ENVO, see the Evaluation Section.

Other European projects target more specific water monitoring data models. This is the case of the ODALA^14^ project that created the ODALA Air & Water application profile^15^. The profile builds on a core module derived from both O&M and the SSN Ontology. In the same direction, an extension of the Smart Applications REFerence Ontology (SAREF) for the water domain (SAREF4WATR^16^) has been implemented as a modular set of versioned ontologies. SAREF4WATR enables semantic interoperability between different Internet of Things (IoT) in order to cover different domains, from water to automotive, eHealth and ageing. Wang *et al*.^17^ describe a knowledge-based approach aiming at water quality monitoring and pollution alerting through the proposed Observational Process Ontology (OPO). Similarly, Diaz *et al*.^18^ presents a three-module water quality ontology that combines numerous standards from different domains to obtain a comprehensive approach to the issue. These standards are, among others, GeoSPARQL^19^, the O&M and SSN cited above, the RDF Data Cube^20^, as well as non-ontological resources associated with standards (WaterML^21^). In the context of the Doce River Project, carried out to support activities to make hydrological-related data available, the Doce water quality ontology^22^ has been carried out in 2019 concerning the impact on the Doce River Basin in Brazil. Furthermore, the European Environmental Agency publishes a Linked Open Data section^23^ that comprises data on water quality monitoring, which enables links with the proposed WHOW-KG.

As far as the health domain is concerned, although it is difficult to find (linked) open data available for the re-use, interesting resources were taken into account when creating the WHOW-KG. In particular, SNOMED Clinical Terms^24^ has been re-used in order to create proper links with our produced controlled vocabulary on infectious diseases. Ontologies have been proposed to solve integration challenges about drug prescription^25^, personalised real-time medical care for specific diseases^26^, human and ecological risks, and smart healthcare systems^27^, though none specifically modelling the data with water quality and consumption, extreme climatic events and infectious diseases.

To the best of our knowledge, although a variety of works can be identified, it is still difficult to get access to a resource capable of linking the water and health domains together as we propose with the WHOW-KG.

## Methods

The WHOW-KG is developed to cope with three selected use cases identified in the context of the WHOW project with domain experts and data providers^28^. The use cases have been gradually developed and fine-tuned using a dual approach:

- Top-down: identifying legislative and policy requirements at the EU and Italian level in the water and health domains;
- Bottom-up: gathering requirements through a co-creation programme where interested stakeholders and users have been involved from the initial phases of the project.

As a result, the use cases are: (i) Contaminants in marine waters (UC1), (ii) Water quality for human consumption (UC2), and (iii) Meteorological extreme events (UC3). The first use case, i.e. Contaminants in marine waters, aims at modelling ontologies and creating linked open data on human exposure to chemicals and biological contaminants in marine waters, ingestion of contaminated fish products, and airborne exposure, such as Ostreopsis Ovata (Ostreopsis Ovata is a well known genus of free-living dinoflagellates found in marine environments that is frequently associated with phenomena of human intoxication). The second use case, i.e. Water quality for human consumption, focuses on generating ontologies and linked open data for modelling and representing quality of surface and ground waters as well as drinking water quality parameters and values, measured by compliance with the EU Directive 2020/2184^29^ on the quality of water intended for human consumption. Finally, the third use case, i.e., Meteorological Extreme events, is about modelling ontologies and linked open data for representing meteorological phenomena, alteration of the hydrological cycle and agriculture industries. More details about the use cases can be found by interested readers in a public deliverable of the project^30^.

### Material

The above-mentioned use cases were defined, along with the identification of relevant open core datasets, through a co-creation programme organised within the WHOW project to which 77 participants actively contributed. The group of co-creators included domain experts, stakeholders, practitioners, and data providers from both public and private organisations located in the EU. The full list of datasets identified can be consulted in a corresponding project deliverable^31^. From this list, we selected high-priority datasets that are currently used for designing and generating the WHOW-KG. The selection was made in compliance with the following criteria: (i) relevance to the use cases; (ii) open licence associated with the dataset; and (iii) data availability for the years spanning from 2018 to 2021. This reference time span is a requirement defined in the project and by the contributors of the co-creation programme. For some datasets, the time period is even longer, starting from 1999 to 2023. Identified datasets are reported in Table 1 with an identifier, short description with a corresponding URL in parentheses for easy access, data format, right holder, supported use case, and number of records (i.e. number of rows and triples for CSV and RDF data sources, respectively). All sources provided by ISPRA are licensed under a CC-BY-4.0 International license, while those provided by ARIA are licensed under a CC0 1.0 license.

**Table 1:** Datasets selected for the creation of the WHOW-KG.

ID	Description	Format	Right holder	Use Case	# of records	ID	Description	Format	Right holder	Use Case	# of records
D1	Observations from tide gauges Network (https://www.mareografico.it/en/data-archive.html)	RDF	ISPRA	UC3	527,423	D10	Analytical data of lake water bodies (https://github.com/whow-project/datasets/raw/refs/heads/main/RML-RULES/hydrography/lakes/hydrography-lakes.tar.gz)	CSV	ARIA	UC2	136 085
D2	Wave motion data (https://www.mareografico.it/en/data-archive.html)	RDF	ISPRA	UC3	135,753	D11	Quality parameters of drinking waters (https://github.com/whow-project/datasets/blob/main/RML-RULES/drinking-water-monitoring/beda-kb7b.csv.zip)	CSV	ARIA	UC3	244,627
D3	Soil consumption indicators (https://groupware.sinanet.isprambiente.it/uso-copertura-e-consumo-di-suolo/library/consumo-di-suolo)	RDF	ISPRA	UC3	1,625,802	D12	Analytical data of underground water (https://github.com/whow-project/datasets/blob/main/RML-RULES/water-monitoring-groundwaters/46wy-4ydd.csv.zip)	CSV	ARIA	UC2	591,390
D4	Repertory of mitigation measures for National Soil Protection (http://www.rendis.isprambiente.it/rendisweb/vistepub.jsp)	RDF	ISPRA	UC3	1,286,758	D13	Water level of the lakes (https://github.com/whow-project/datasets/blob/main/RML-RULES/water-monitoring-lakes/Altezza_laghi.csv)	CSV	ARIA	UC2	57,924
D5	Bathing water quality indicators (https://sdi.eea.europa.eu/catalogue/srv/api/records/5d9a4d94-511a-486d-afbb-4f01e5c73e23?language=all)	CSV	ISPRA	UC1	5,529	D14	Description of Health Protection Agencies (https://github.com/whow-project/datasets/blob/main/RML-RULES/ats/ats.tar.gz)	CSV	ARIA	UC2	8
D6	Ostreopsis ovata (https://github.com/whow-project/datasets/raw/refs/heads/main/RML-RULES/ostreoptis-monitoring/ostreoptis.tar.gz)	CSV	ISPRA	UC1	1,222	D15	Hospital stay and access rates (https://github.com/whow-project/datasets/blob/main/RML-RULES/hospitalisation-monitoring/rzni-6n8h.csv)	CSV	ARIA	UC2	17,412
D7	Pesticides in waters (https://w3id.org/italia/env/ld/pest/dataset)	RDF	ISPRA	UC2	89,635,028	D16	Drug consumption rate (https://github.com/whow-project/datasets/blob/main/RML-RULES/drug-consumption-monitoring/2mr3-henm.csv)	CSV	ARIA	UC2	7,200
D8	Administrative places in Italy (https://w3id.org/italia/env/ld/place/dataset)	RDF	ISPRA	UC1-3	355,886	D17	Infectious diseases rates by sex and age (https://github.com/whow-project/datasets/blob/main/RML-RULES/infectious-disease-monitoring/infectious_diseases.csv)	CSV	ARIA	UC2	11,436
D9	Analytical data of river water bodies, including flow rate (https://github.com/whow-project/datasets/raw/refs/heads/main/RML-RULES/hydrography/rivers/hydrography-rivers.tar.gz)	CSV	ARIA	UC2	1,060,320	D18	Weather observations and stations	CSV	ARIA	UC1-2	4,627,443

Further information about the datasets' source is in the Supplementary Information and can be consulted in the project deliverable^31^.

### Ontology Modelling

The methodology we used for constructing the ontologies and linked open data part of the WHOW-KG is inspired by the one defined in Carriero *et al*.^32^ and relies on eXtreme Design^33^ (XD) for ontology modelling. XD emphasises the reuse of ontology design patterns^34^ (ODPs) into an iterative and incremental process. More interestingly, XD is a collaborative methodology that fosters the cooperation among multiple actors with different roles (e.g. knowledge engineers, domain experts, etc.) to make sure all the modelling requirements are first captured and then effectively covered. Hence, we opted for XD since it fits our collaborative setting based on the co-creation programme. Furthermore, there is evidence in the literature^35^ that the reuse of ODPs (i) speeds up the ontology design process, (ii) eases design choices, (iii) produces more effective results in terms of ontology quality, and (iv) boosts interoperability. In this section, we will describe how the ontology design and the Linked Open Data (LOD) production were carried out with XD.

**Fig. 1:**
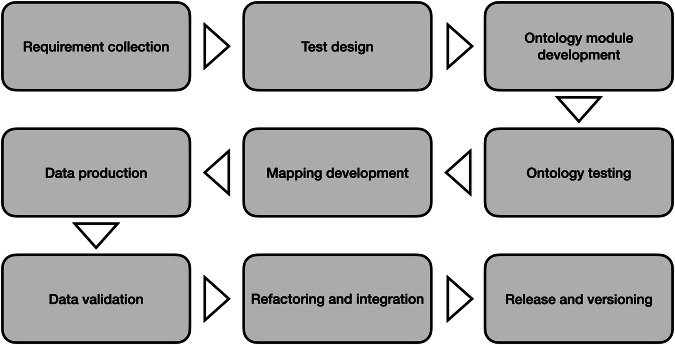
Methodology implemented for constructing the WHOW-KG.

Figure 1 shows the methodology implemented for constructing the WHOW-KG.

#### Ontology design

As Fig. 1 shows, the XD ontology design process begins with the *requirement collection*, followed by the activities of *test design*, *ontology module development*, and *ontology testing*.

- **Requirement Collection:** The requirement collection activity aims at eliciting the requirements as *competency questions*^36^ (CQs). CQs are natural language questions conveying the ontological commitment expected from a knowledge graph and drive both ontology modelling and validation. In fact, on the one hand CQs are a mean for ontology development. On the other, they can be converted to formal queries in order to assess the effectiveness of the resulting KG to cope with the requirements.
- **Test Design:** We implemented the validation into the *ontology testing* activity. This was done by converting CQs to SPARQL and executing the latter as unit tests with sample data following the solution defined in Carriero *et al*.^37^.
- **Ontology Module Development:** The ontology development we applied is modular (cf. activity named ontology module development), allowing us to generate a set of networked ontologies. Each ontology of the network is a separate module designed with the purpose of minimising coupling with other ontology modules and maximising the internal cohesion of its conceptualisation. The re-use of external ontologies and ODPs was done by applying both the direct and indirect approach^38,39^.
- **Re-use of External Ontologies and Ontology Design Patterns (ODPs):** Direct re-use is about embedding individual entities or importing implementations of ODPs or other ontologies in the network, thus making it highly dependent on them. Instead, indirect re-use is about applying relevant entities and patterns from external ontologies as templates, by reproducing them in the ontologies of the network and providing possible extensions. We opted for direct re-use in case of widely adopted vocabularies, such as SKOS^40^, the Time ontology available in the Italian national catalog of semantic assets for public administrations^41^, aligned with the W3C time ontology^42^, and the top-level^43^ (TOP) and environmental monitoring facilities^44^ (EMF) ontologies of the Linked ISPRA project^45^. TOP is used as a top-level ontology that provides general concepts and relations, whilst EMF provides core domain concepts and relations for modelling environmental monitoring data. On the contrary, we opted for the indirect approach for re-using patterns and to support interoperability with other pertinent ontologies, e.g. SSN/SOSA^10,46^. The latter case was realised by means of alignments axioms, such as rdfs:subClassOf and owl:equivalentClass available in the ontology files.

#### Linked Open Data production

Once the ontology network is modelled, the next steps aims at populating the KG with Linked Open Data gathered from the input data sources (cf. Table 1). The LOD production was performed by means of declarative mappings (i.e. the activity *mapping development*). That is, we defined a set of mapping rules specified with RDF Mapping Language^47^ (RML), which extends the W3C-standardised mapping language R2RML^48^ for mapping to RDF any possible type of structured data source. All the RML mapping rules we defined are published under a CC-BY-4.0 license on Zenodo^49^ and maintained on GitHub^50^ for versioning. These mappings were processed with pyRML^51^, a lightweight Python engine for processing RML files designed and implemented in the context of the WHOW project. Data validation was then performed by using the same SPARQL queries derived from CQs as unit tests. It is worth noticing that unit testing was performed on real data. The activities related to data production are meant to be executed in a decentralised and distributed fashion in which different data providers might use their own data and RML mapping rules independently.

## Results

### Ontology Network

**Fig. 2:**
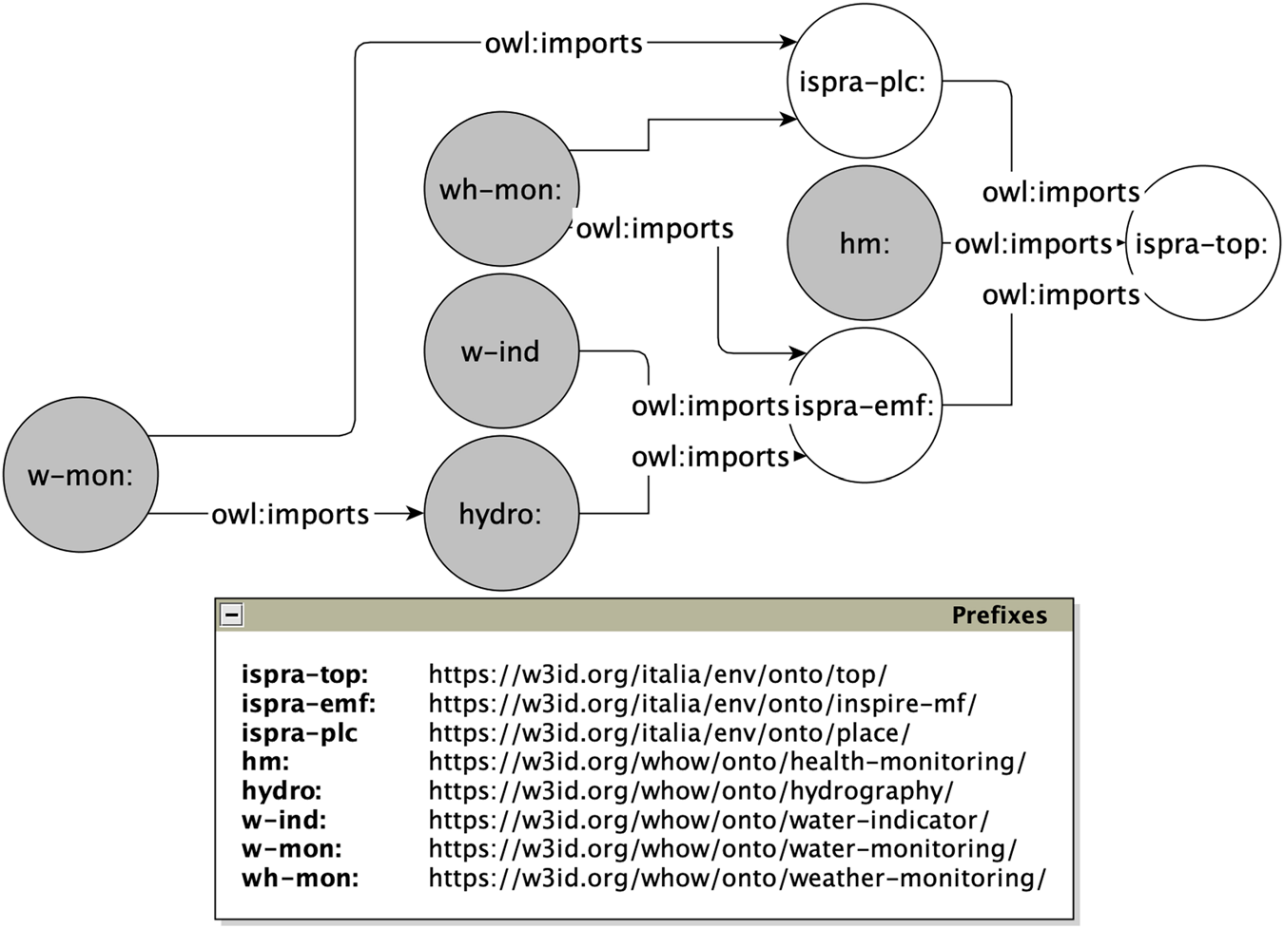
The WHOW ontology network.

The WHOW ontology network consists of 8 ontology modules that formalise a shared language for modelling, representing, exchanging, and interacting with data in the WHOW-KG. In Fig. 2 each ontology is represented as a circle, whilst the arrows represent owl:imports axioms among the ontologies. The ontologies represented as white circles are external ontologies we re-used with the direct approach. The ontologies represented as gray circles are the novel contributions. The base namespace defined for novel ontologies is https://w3id.org/italia/whow/onto/. From this base namespace each module defines its pertinent namespace following the associations reported in the table of prefixes in Fig. 2. Table 2 reports core metrics about the ontology network, which is: (i) under version control on GitHub^52^; (ii) shared on Zenodo^53^ with a CC-BY 4.0 International License; and (iii) findable on Linked Open Vocabularies^54^. Each ontology module is further detailed in the following subsections.

**Table 2:** Statistics of the ontology network.

Metric	Value	Metric	Value
Axioms	2,712	SubObjectPropertyOf axioms	141
Classes	126	Inverse object properties	62
Object properties	168	Transitive object properties	10
Datatype properties	21	Declared property domains	162
DL expressivity	SRIQ(D)	Declared property ranges	160
SubClassOf axioms	275	Property chains	6
Disjoint classes	23	Annotation assertions	1,491

#### Hydrography module

**Fig. 3:**
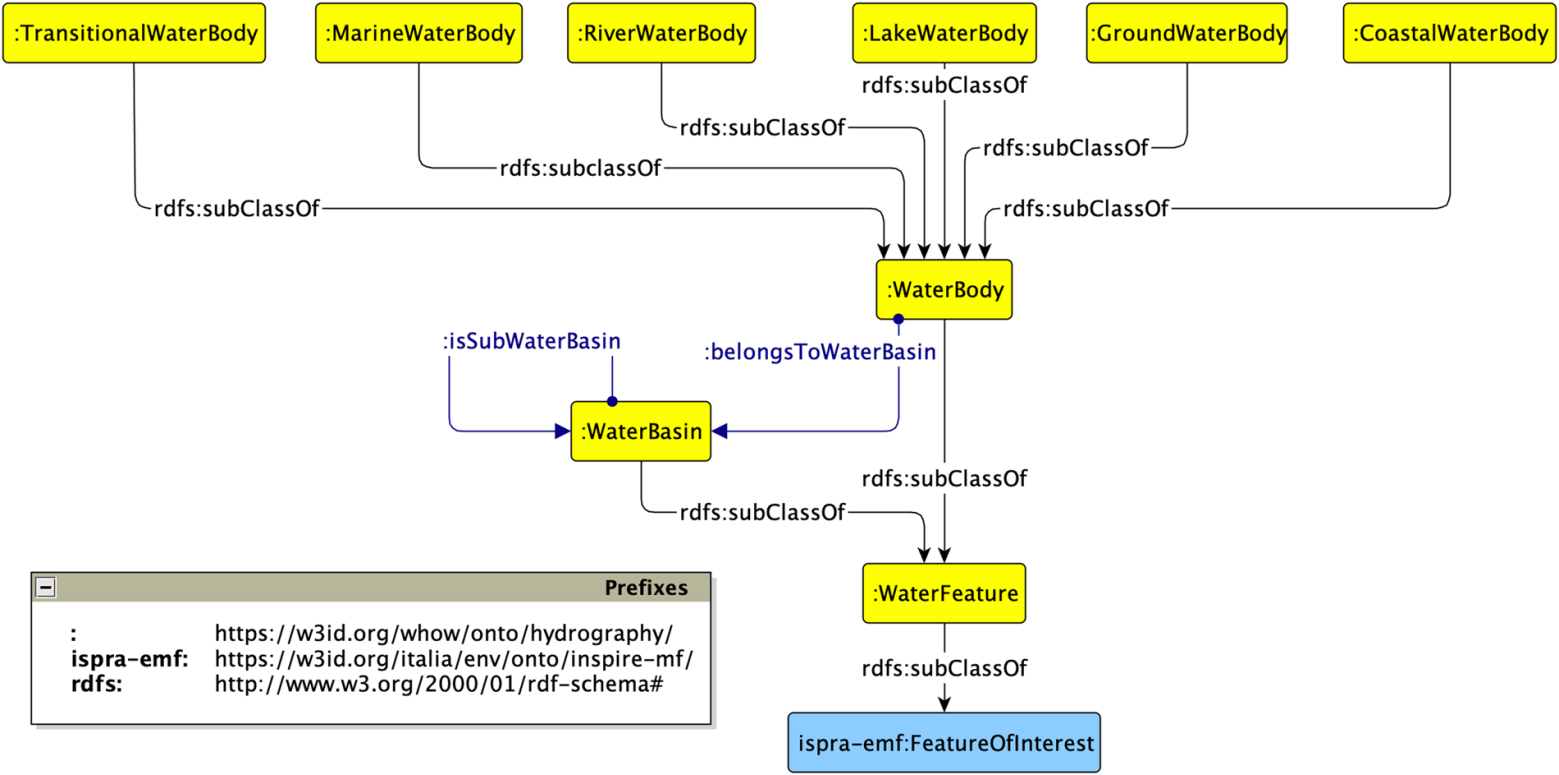
The Hydrography ontology.

The *Hydrography* ontology is identified by the prefix hydro: (The prefix hydro: stands for the namespace in^55^). It represents a general-purpose hydrological taxonomy compliant with the European Directive 2000/60/EC^29^ in the field of water policy. The Hydrography ontology is depicted in Fig. 3 using Graffoo^56^ as the reference notation. Blue rectangles indicate classes re-used with the direct approach from external ontologies. Instead, yellow rectangles indicate new classes we defined in the scope of the Hydrography ontology. The top-level class is hydro:WaterFeature, a subclass of the ISPRA ontology ispra-emf:FeatureOfInterest with hydro:WaterBasin and hydro:WaterBody as subclasses. A hydro:WaterBody further specialises into a number of subclasses defining a clear classification among the different types of water bodies. Those subclasses are hydro:TransitionalWaterBody, hydro:MarineWaterBody, hydro:RiverWaterBody, hydro:LakeWaterBody, hydro:GroundWaterBody, and hydro:CoastalWaterBody. In this ontology we reused the PartOf ODP^57^ for expressing parthood relations between water basins (cf. the object property hydro:isSubWaterBasin).

#### Water Monitoring module

**Fig. 4:**
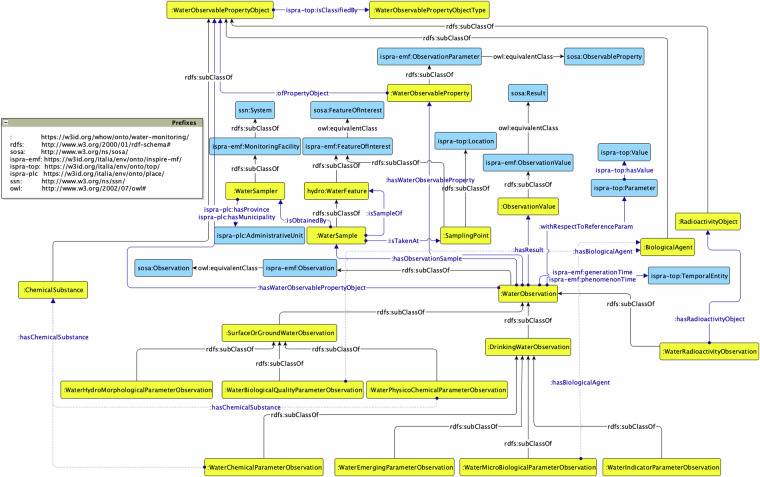
The Water Monitoring ontology.

The *Water Monitoring* ontology is identified by the prefix w-mon: (The prefix w-mon: stands for the namespace in^58^. It allows our data to represent observations related to the quality of water courses, such as chemical and biological substances found in water bodies. The requirements for the representation of water observations are defined according to the data provided by the data providers involved in the project and the standards and directives in terms of observations and water-related assessments. For what concerns the representation of water observations, it is possible to refer to European directives: (i) those deriving from taxonomies from European Directive 98/83/CE (and subsequent ones)^59^, confirmed by the Italian Ministry of Health (Water quality parameters published by Italian Ministry of Health^60^), concerning parameters of the waters for human consumption, and (ii) those deriving from the European Directive 2009/90/EC^59^, concerning parameters of surface waters. Thus, water quality monitoring requires the integration of heterogeneous types of both observations and observation objects derived from samplers. As a result, in the ontology (cf. Figure 4), the class *w-mon:WaterObservation* is further specialised by w-mon:DrinkingWaterObservation, w-mon:SurfaceOrGroundWaterObservation, and w-mon:RadioActivityObservation, which are, in turn, further divided into subclasses based on the specific parameter being observed. In fact, the observations that have as an object a microbiological agent or a chemical substance, monitor it through its concentration in the water. On the contrary, observations on properties of water, such as hardness, density or pH, do not imply the presence of an object being observed since no chemical substance or microbiological agent is implied there. The ontology follows the Stimulus-Sensor-Observation Ontology Design Pattern (SSO ODP)^61^, which is a standard for the Infrastructure for Spatial Information in Europe^62^, and the Specimen model of ISO 19156:2011^63^, which outlines the properties of sampling process features.

#### Water Indicator module

**Fig. 5:**
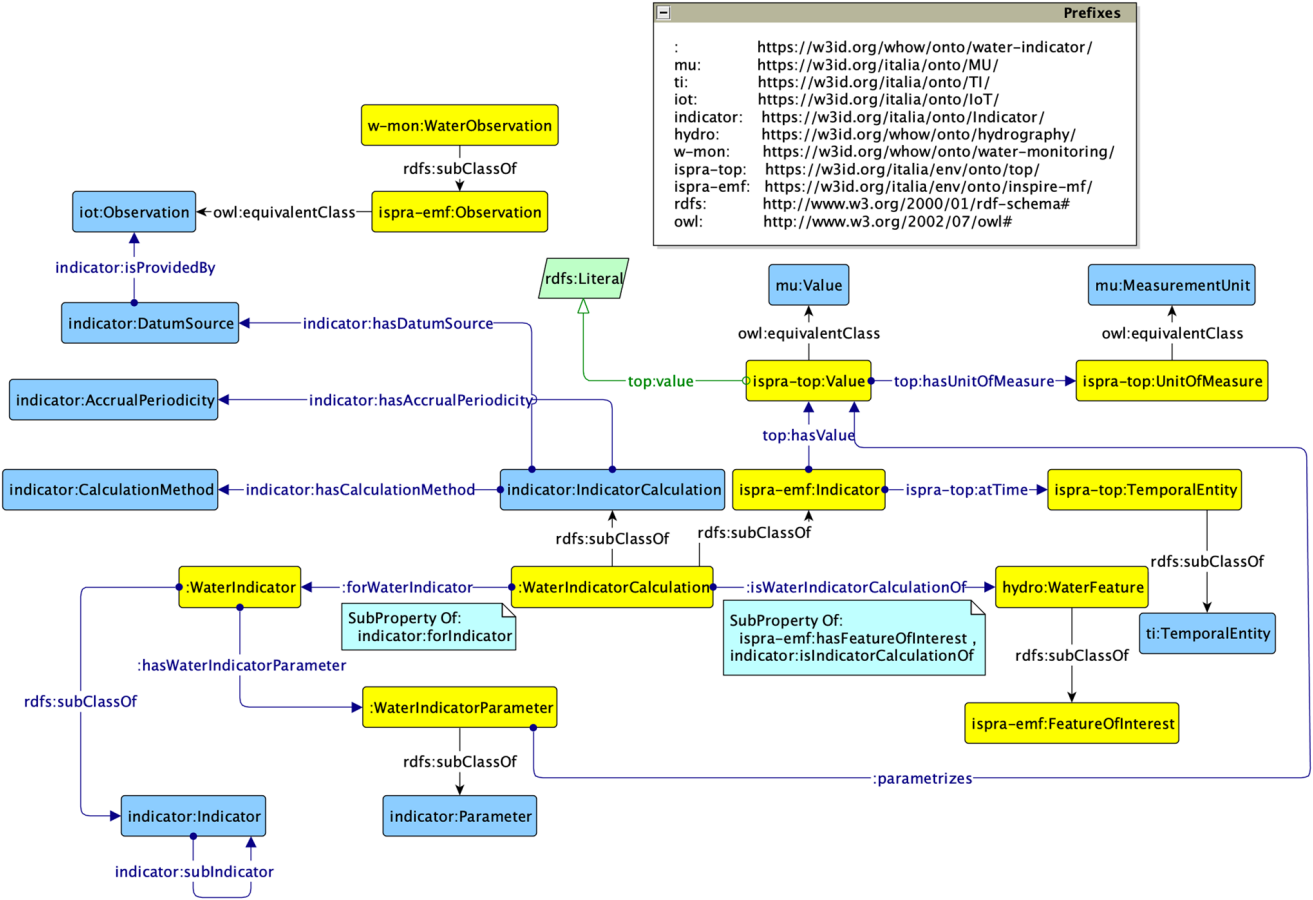
The Water Indicator ontology.

The *Water Indicator* ontology, with prefix w-ind: (the prefix w-ind: stands for the namespace in^64^), re-uses the Indicator ontology design pattern^65^ defined in OntoPiA^66^, which is the Italian national network of ontologies and controlled vocabularies. This pattern is re-used to address indicators and metrics for calculation of water quality indicators. As shown in Fig. 5, the indicators can be bathing water quality classes or indicators of lakes’ chemical status.

#### Weather Monitoring module

**Fig. 6:**
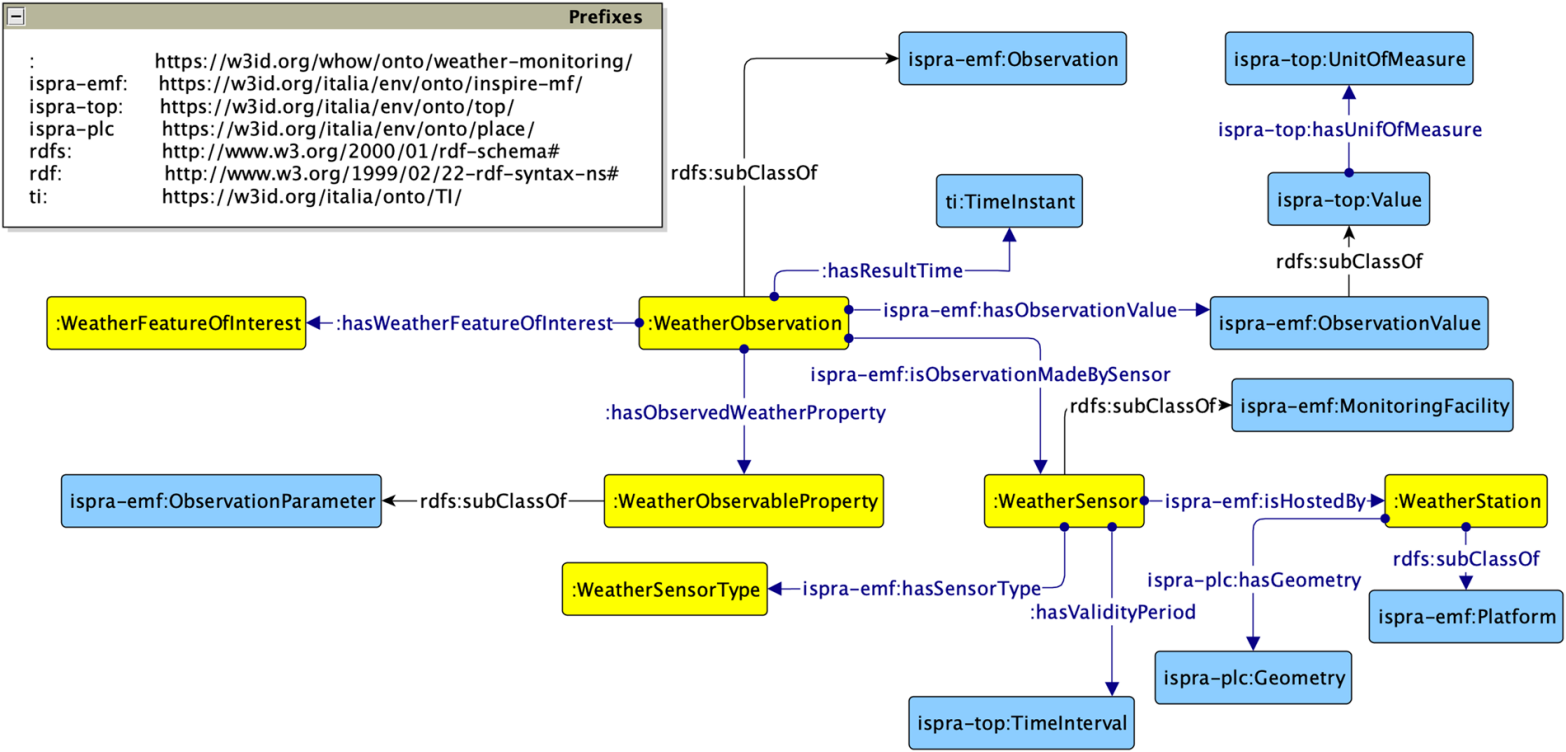
The Weather Monitoring ontology.

Similarly to the Water Monitoring module, the *Weather Monitoring* ontology, with prefix wh-mon: (The prefix wh-mon: stands for the namespace in^67^ cf. Figure 6), has its focus on a wh-mon:WeatherObservation related to a wh-mon:WeatherFeatureOfInterest (either ground-level soil, air, wind, snow or rainfall), wh-mon:WeatherObservableProperty and wh-mon:WeatherSensor hosted by a wh-mon:WeatherStation. It reuses the ISPRA ontology network to model observations and related properties. This module addresses the requirement to represent weather observations that could serve as a basis to derive information on extreme events monitoring and prediction, such as rainfalls and snow levels.

#### Health Monitoring module

**Fig. 7:**
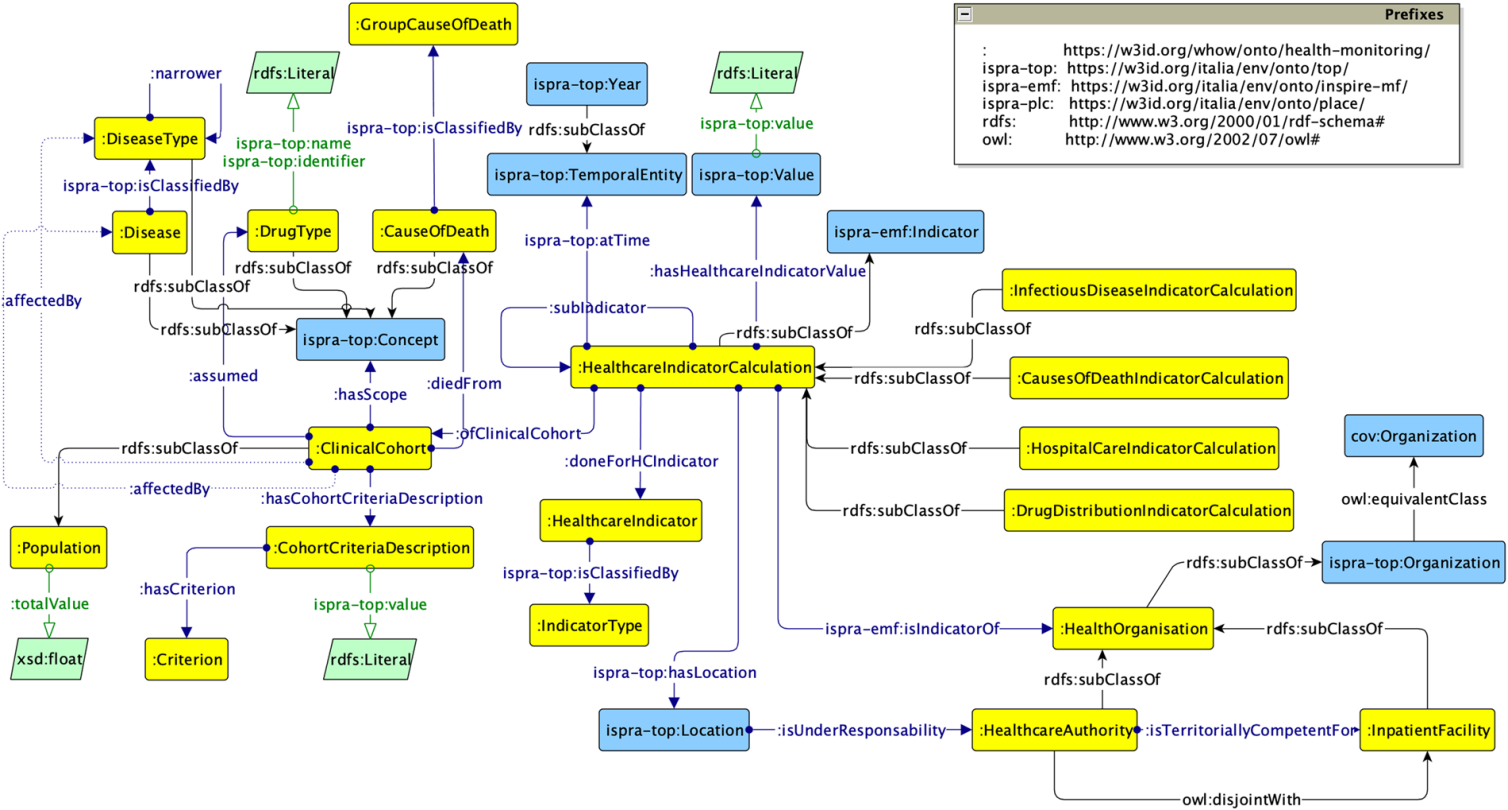
The Health Monitoring ontology.

Finally, the *Health Monitoring* ontology, whose prefix is hm: (The prefix hm-mon: stands for the namespace in^68^) reuses the OntoPiA Indicator ontology and focuses on the representation of health indicators coming from regional healthcare facilities. Examples include drug distribution rates and hospital accesses according to disease code and healthcare facility involved (cf. Figure 7). Different types of hm:HealthcareIndicatorCalculation are defined, based on the type of indicator they describe, i.e. infectious disease rate, death rates related to diagnosis, average hospital stay and drug distribution. The indicator calculation also refers to a class associated with a statistical dimension, i.e. hm:ClinicalCohort, which specifies the population referred to as defined by a number of criteria, that is hm:CohortCriteriaDescription, such as age and gender. By reusing the ispra-top: ontology, it is also possible to model the health agency that supervises a specific area.

### Linked Open Data

We produced the Linked Open Data from two data providers, i.e. ISPRA and ARIA, as reported in Table 1 by executing the RML mappings as described in the Methods Section. Accordingly, we generated two linked open datasets, that is, the one from the data provided by ISPRA and the other from the data provided by ARIA. The ownership of and rights on the generated linked datasets along with their corresponding maintenance effort is kept by the data providers. This fits the requirement of WHOW to create and maintain a knowledge graph following a decentralised and distributed paradigm. Furthermore, this decentralised ecosystem has also the potential to be supported through the publication of data within Solid pods^69^ that support SPARQL, ensuring transparency and accountability of the data collection process among data providers^70^.

In this scenario, new data providers might publish their data as linked open data compliant with the WHOW ontology network by using their own defined persistent URIs and setting up their own SPARQL endpoint, thus maximising the sustainability of the WHOW-KG. With this regards, ISPRA identified https://w3id.org/italia/env/ld/ as its reference namespace for persistent URIs. Accordingly, the pattern https://w3id.org/italia/env/ld/*{type}/{id}* was applied for producing RDF resources, where *{type}* and *{id}* are placeholders for an entity type (e.g. water-sample) and its local identifier (e.g. 45.60555-13.72195), respectively. The RDF data produced by ISPRA can be queried through a dedicated SPARQL endpoint^71^ and are available as a single dump on Zenodo^72^ for download under the CC-BY 4.0 International License. Similarly, ARIA identified https://w3id.org/italia/lombardia/data/ as its reference namespace for persistent URIs. Also in this case, the pattern https://w3id.org/italia/lombardia/data/*{type}/{id}*was applied for producing RDF resources with the same rationale as before. Again, the RDF data produced by ARIA can be queried via SPARQL^73^ and are available on Zenodo^74^ under a CC0 License. Finally, three controlled vocabularies were produced from the data provided by ARIA. These vocabularies provides term definitions for: (i) chemical substances^75^; (ii) diseases^76^; and (iii) water indicators^77^. In the case of controlled vocabularies we opted for a namespace not depending on the specific data provider, i.e. https://w3id.org/whow/controlled-vocabulary/. This namespace was used for producing RDF resources by applying the pattern https://w3id.org/whow/controlled-vocabulary/*{name}/{id}*, where *{name}* and *{id}* are placeholders for the vocabulary name (e.g. chemical-substances) and the local identifier of a term (e.g. cas-102851-06-9), respectively. The controlled vocabularies are available on Zenodo^78^ under the CC-BY 4.0 International License and can be queried via SPARQL^79^. The WHOW-KG counts of 152,246,711 triples in the linked dataset generated by ISPRA, 113,190,050 triples in the linked dataset generated by ARIA, and 16,350 triples available in the controlled vocabularies, thus generating a total of 265,453,111 RDF triples. Information about the datasets with corresponding number of triples is shown in Table 3. Whenever possible, entities in linked data and concepts in the controlled vocabularies were linked to external vocabularies and linked datasets using equivalence axioms, i.e. owl:sameAs, defined in the RML mappings. Specifically, the concepts in the controlled vocabulary for classifying diseases (https://w3id.org/whow/controlled-vocabulary/diseases) are linked to clinical terms in SNOMED^24^, a comprehensive, standardized clinical healthcare terminology used worldwide. Similarly, the concepts in the controlled vocabulary for classifying chemical substances (https://w3id.org/whow/controlled-vocabulary/chemical-substances) are linked to entities in Wikidata^80^, a free, collaborative, multilingual knowledge base, and to LEO (https://opendata.leo-italy.eu/portale/home), a linked open dataset on livestock and the environment. Finally, entities in the linked datasets, such as places and unit of measures were linked to Wikidata and to codes maintained by the World Meteorological Organisation (https://codes.wmo.int/).

**Table 3:** Datasets part of WHOW-KG with corresponding number of triples.

ID	Description	Triples	ID	Description	Triples
D1	Observations from tide gauges Network	527,423	D10	Analytical data of lake water bodies	1,409,794
D2	Wave motion data	135,753	D11	Quality parameters of drinking waters	2,734,537
D3	Soil consumption indicators	57,528,925	D12	Analytical data of underground water	5,959,926
D4	Repertory of mitigation measures for National Soil Protection	2,290,973	D13	Height of the lakes	57,924
D5	Bathing water quality indicators	1,669,340	D14	Description of Health Protection Agencies	385
D6	Ostreopsis ovata	103,383	D15	Hospital stay and access rates	483,843
D7	Pesticides in waters	89,635,028	D16	Drug consumption rate	151,489
D8	Administrative places in Italy	355,886	D17	Infectious diseases rates by sex and age	464,532
D9	Analytical data of river water bodies, including flow rate	10,638,603	D18	Weather observations and stations	91,772,860

#### Usage examples

The WHOW-KG can answer a variety of questions that encompass the three use cases reported in the Section Methods. For example, it would be possible to retrieve the contaminants recorded in a water body used for human consumption (UC2). A possible question is the following: *What is the concentration of reactive silicates observed for the Lake Endine over time?* This question can be converted into the following SPARQL query:


SELECT?obs?time?value?unitWHERE{?obs a wm:WaterPhysicoChemicalParameterObservation;emf:hasFeatureOfInterest data-lwb:endine;wm:hasWaterObservableProperty data-op:1344-09-8;wm:hasResult/top:value?value;wm:hasResult/top:hasUnitOfMeasure/top:name?unit;emf:generationTime/top:time?time.}ORDER BY?time


**Fig. 8:**
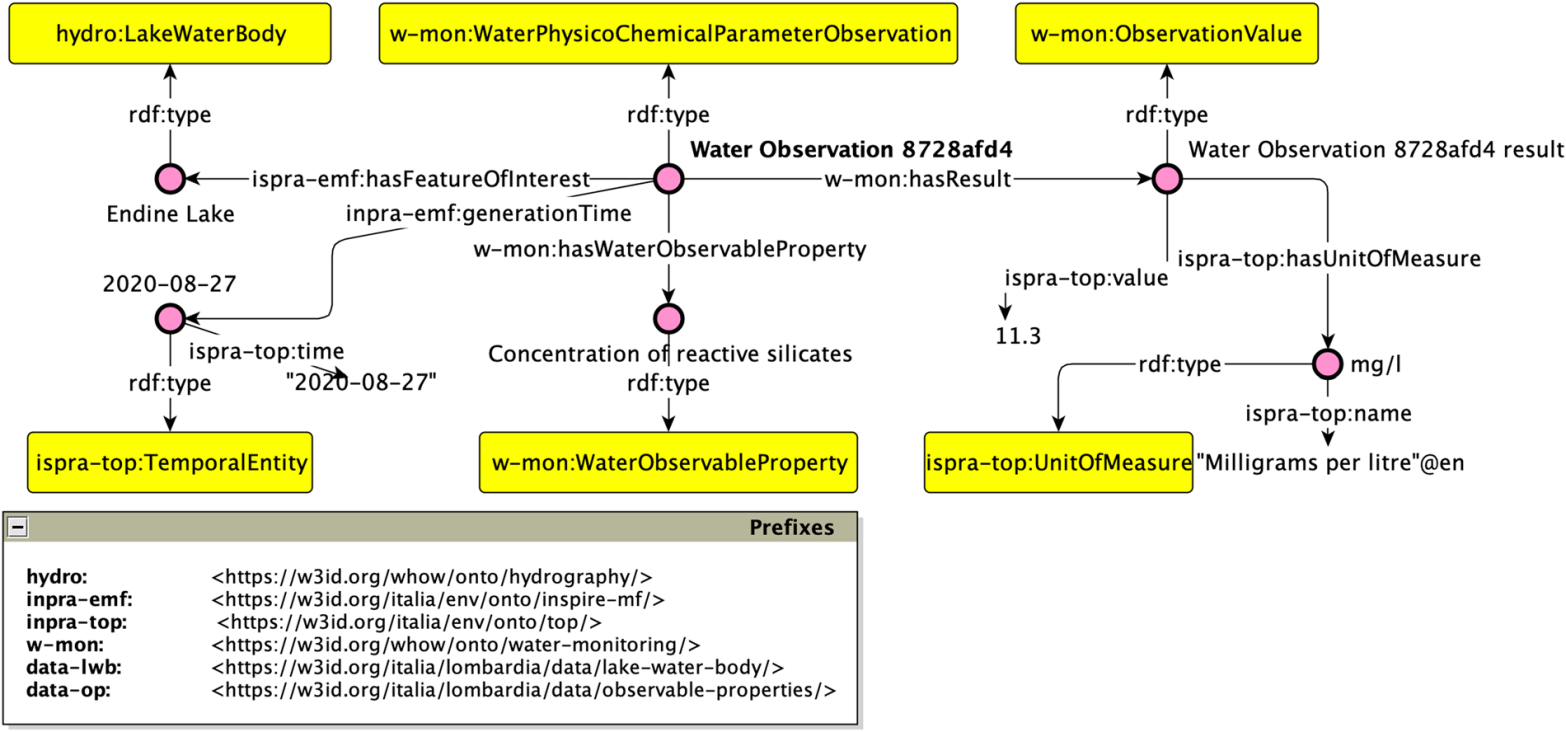
The RDF graph answering the question *What was the concentration of reactive silicates observed for the Lake Endine over time?*.

An exemplification of the result set is represented by the RDF graph depicted in Fig. 8. A full result set can be obtained by executing the previous SPARQL query on the endpoint exposed by ARIA. In this graph the entity Water Observation 8728afd4 is a wm:WaterPhysicoChemicalParameterObservation as defined by the Water Monitoring module (cf. Ontology Network Section) and is connected to: (i) a feature of interest, which is the Lake Endine typed as hydro:LakeWaterBody; (ii) an observation result, i.e. Water Observation 8728afd4 result typed as a w-mon:ObservationValue, which is associated with a specific literal value (i.e. “11.3”) and a unit of measure (i.e. mg/l); (iii) a generation time for the observation which is the ispra-top:TemporalEntity identified by the entity 2020-08-27 with its corresponding literal value.

Another possible question related to UC1 might be focused on retrieving the observations of biological agents like the Ostreopsis Ovata in marine water bodies. An example of this case is the question: *What are the biological agents with their associated concentration recorded over time for the Adriatic sea?* This question can be converted into the following SPARQL query:


SELECT?obs?bioagent?link?timeWHERE{?obs a w-mon:WaterBiologicalQualityParameterObservation;ispra-emf:generationTime?time;ispra-emf:hasFeatureOfInterest data:adriatic-sea;w-mon:hasResult?res;w-mon:hasBiologicalAgent?bioagent.OPTIONAL{?bioagent owl:sameAs?link}}


**Fig. 9:**
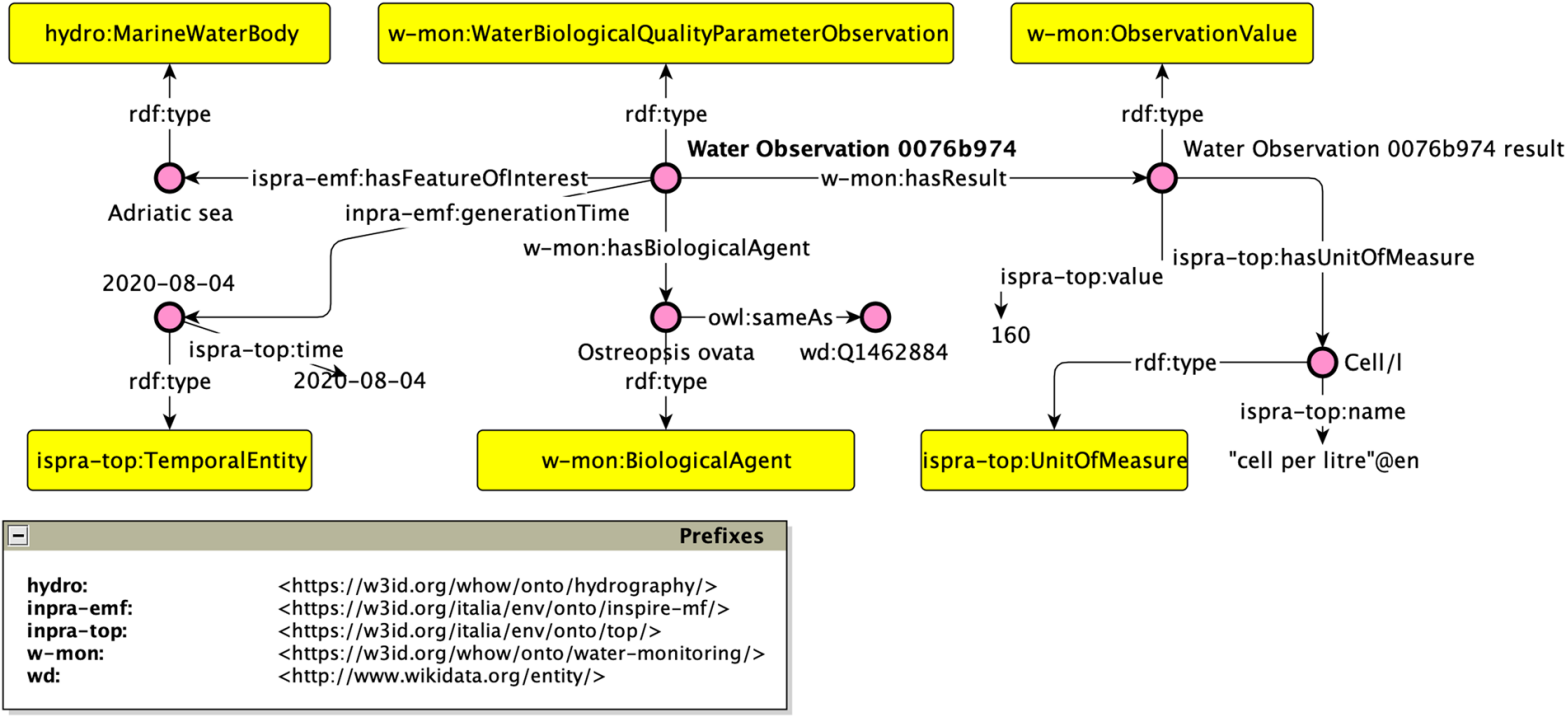
The RDF graph answering the question *What were the biological agents with their associated concentration recorded over time for the Adriatic sea?*.

An exemplification of the result set is represented by the RDF graph depicted in Fig. 9. Such a graph is derived from the execution of the previous SPARQL query against the endpoint exposed by ISPRA^71^. In this case, the entity Water Observation 0076b974 is a w-mon:WaterBiologicalQualityParameterObservation and is connected to: (i) a feature of interest, which is the Adriatic sea typed as hydro:MarineWaterBody; (ii) an observation result, i.e. Water Observation 0076b974 result typed as a w-mon:ObservationValue, which is associated with the value 160 cell per litre; (iii) a generation time for the observation which is the ispra-top:TemporalEntity identified by the entity 2020-08-04 with its corresponding literal value; (iv) the biological agent represented by the Ostreopsis ovata, which is typed as w-mon:BiologicalAgent.

The observational data on water quality can be linked to data about the average rate of hospitalisation for a same geographical area that reports diseases. A possible example of SPARQL query that addresses this case is presented below and provides the average accesses in the hospitals located in Lombardy, Italy, associated with time and disease recorded. This query can be executed against the endpoint exposed by ARIA^73^. Additional examples for all use cases can be found on GitHub^50^.


SELECT?calc?value?disease?yearWHERE{?calc a hm:HospitalCareIndicatorCalculation;hm:doneForHCIndicator indicator:average-total-number-access;hm:hasHealthcareIndicatorValue/ispra-top:value?value;ispra-top:atTime/ispra-top:year?year;hm:ofClinicalCohort/hm:affectedBy?disease}


## Evaluation

In this section, we report the result of the evaluation we carried out for the WHOW-KG.

### Ontology network evaluation

In this section, we report the results of the assessment of the ontology network with respect to (i) graph centrality measures and (ii) terminological coverage computed by taking into account domain-specific documents about Water Directives.

#### Concept centrality evaluation

To evaluate the relevance of nodes in complex networks, centrality, focusing on degree centrality, eigenvector centrality, and betweenness centrality, are common evaluation measures for knowledge graphs^81^. These metrics highlight the number of connected neighbours, the influence of nodes, and their participation in the shortest paths, respectively. By identifying ontology-linked nodes with the highest centrality, we can assess what are the most important concepts^82^. We consider only the WHOW ontology network classes. More information can be found in the Supplementary Information complementing this paper.

**Degree centrality**. The nodes with the highest degree centrality values represent concepts from all the ontology modules, with a specific focus on water. In fact, the most central node is w-mon:WaterObservation, the second being h-mon:HealthcareIndicatorCalculation, followed by w-mon:WaterSample, w-ind:WaterIndicatorCalculation and hydro:WaterFeature.

**Eigenvector centrality**. The eigenvector centrality of a node indicates the number of nodes it directly connects to, along with those indirectly connected through them, and so on throughout the network. This metric measures a node’s influence within the network relative to its connections with all other nodes across the entire network. In our case, w-mon:WaterObservation is the node with the most influence, followed by w-mon:WaterSample, w-mon:WaterObservablePropertyObject, w-mon:DrinkingWaterObservation and w-mon:BiologicalAgent. As all of these are from the Water Monitoring Module, this result attests the centrality of the module in relating the concepts in the WHOW-KG.

**Betweenness centrality**. Betweenness centrality quantifies how frequently a node lies along the shortest path between two distinct nodes. A node with high betweenness centrality acts as a bridge across numerous shortest paths between different nodes, positioning itself either centrally within the network or within a significant cluster. The higher one is w-mon:WaterObservablePropertyObjectType, which indeed serves as a bridge between an observation and the type of the object it pertains to, followed by w-mon:WaterObservation, h-mon:HealthcareIndicatorCalculation, wh-mon:WeatherSensorType and wh-mon:WeatherObservation.

#### Terminological coverage evaluation

Automated evaluation of an ontology network includes the analysis of its ability to cover domain-specific terminology with respect to other available ontologies^83^. In this sense, the terminological coverage is seen as an ontology alignment problem between a selected, automatically extracted vocabulary and the target ontologies. There are multiple ways to extract such a vocabulary: starting from a predefined set of documents, in Carriero *et al*.^84^, this has been done with Rapid Automatic Keyword Extraction (RAKE); Demaidi and Gaber used a custom keyword extraction tool, TONE^85^, while Zaitoun *et al*.^86^ focused on a domain-tuned named-entity-recognition-model. As LLMs have been proved to absorb, infer, and reproduce information from self-supervised training on large quantities of text^87^, we expand the approach of Carriero *et al*.^39^ not only to two different RAKE algorithms (nltk^88^ and RAKE-keyword^89^), but also to three different LLMs: GPT (in the Chat, version 4), LLaMa (version llama-3-sonar-large-32k-online), and Claude (version 3-haiku). In our case, we have taken as a reference the Directive 2000/60/EC establishing a framework for Community action in the field of water policy^59^. The ontologies to compare the results with have been selected basing on the domain relatedness and on the availability of the ontology network, along with descriptions, labels, or comments of it, online. They are DOCE^22^, aimed to produce and analyse water quality data of the Doce River Basin, and SAREF4WATR^16^, the SAREF extension for the water domain. Furthermore, we included the ENVO ontology^13^, a general-purpose representation of environments, environmental processes, ecosystems, habitats, and related entities.

The results in Table 4 show that the WHOW Ontology Network exhibits broader terminological coverage compared to the other two ontologies related to water monitoring, i.e. DOCE and SAREF4WATR, which represents our core domain of interest. Conversely, ENVO provides a slightly better terminological coverage than WHOW for most of the measurement applied. Nevertheless, this result is not recorded consistently as in cases where GPT is used, WHOW outperforms ENVO in terms of terminological coverage. This is because ENVO is a large, general-purpose ontology in the context of the environmental domain with a high likelihood of containing keywords relevant to the water directive. In contrast, the WHOW Ontology Network is specifically designed to address selected use cases, focusing on water monitoring while also serving as a foundation for the knowledge graph with integrated data. Therefore, this automated evaluation performs differently at various levels of granularity. Further details on the evaluation can be found in the Supplementary Information section.

**Table 4:** Measurement Results.

Measurement	WHOW Ontology Network	DOCE	SAREF4WATR	ENVO
Rake-nltk	7.22%	3.73%	5.38%	18.79%
Rake-keyword	6.28%	3.17%	4.01%	13.92%
GPT	69.93%	39.89%	54.85%	58.15%
LLaMa	10.03%	10.41%	0%	16.24%
Claude	58.40%	44.54%	38.66%	81.09%

### Linked Open Data evaluation

We also evaluate the Linked Open Data part of WHOW-KG with respect to its adherence to FAIR principles and other measures that show compliance to current data quality standards. Furthermore, we show the added value of our data by comparing the results of selected queries to other knowledge bases.

#### FAIR principles evaluation

To evaluate the quality of the datasets part of WHOW’s knowledge graph, WHOW adheres to the FAIR Data Maturity Model^90^ which, we claim, guides the openness of all the data in every application domain, not only in open science.

We chose specific indicators from the FAIR Data Maturity Model that are pertinent to both assessing the original quality of the data sets used in WHOW and ensuring the quality standards established by the project activities. This assessment was derived from the attribute of a scale of relevance, from “essential” to “not relevant”. In addition to these indicators, our data quality framework also incorporates elements from the ISO/IEC 25012 and 25024 standards^91^, which define additional data quality characteristics.

Not all indicators from the FAIR model have been implemented; the project has prioritized only those deemed “essential” and “important”, which are the top two categories in the project’s relevance hierarchy. This selective approach is motivated by the desire to provide a data quality framework that is both easy to implement for data producers and consumers and robust enough to support the publication of high-quality open datasets.

Furthermore, we designed a framework to perform assessment by a Validation Manager^92^. In our case, data catalogues from the two providers, ISPRA and ARIA, are harvested by the Italian National Data Catalogue. This national catalogue is then harvested by the European Data Catalogue, which performs the Metadata Quality Assessment (MQA) for each national data catalogue using the MQA tool^93^. Upon passing a SHACL-based validation, the Validation Manager proceeds with a MQA as outlined by the official portal for European data. This assessment evaluates various metadata indicators across five dimensions: Findability, Accessibility, Interoperability, Reusability, and Contextuality. The rating system and the corresponding rating categories are structured following the same methodology as the European Data Portal^93^, which to specific dimensions attributes metrics and weights. The final rating is a sum of each of the dimensions’ scores, which correspond to a final rating label from Excellent to Bad.

The results from this assessment, formalised by the official portal for European data and computed using the metadata validation tool of the WHOW Toolkit^94^, have been recorded. All datasets provided by ISPRA and their RDF distributions achieved a global rating of 400, classified as excellent based on the MQA scoring range. For ARIA, the global rating achieved is 390, classified as excellent as well.

#### Return results of queries of the KG with respect to Wikidata and ChatGPT

To explore the extent of information richness in the WHOW-KG, we assessed specific queries to compare the results retrieved from the linked data with the data contained in other knowledge bases, such as Wikidata (where applicable) and ChatGPT.

In line with the FAIR principles, being findable and accessible, the WHOW-KG should offer added value by returning useful, detailed information not available elsewhere. Therefore, we selected four Competency Questions to translate into queries for the ARIA SPARQL Endpoint^73^:

1. *How many water bodies are there in Lombardy, and what are their names and types?* This question underscores the importance of having a taxonomy of water bodies, as well as coverage of specific rivers in the Lombardy Region.
2. *What is the concentration of reactive silicates observed for Lake Endine?* This query focuses on cross-referencing information about water bodies and chemical substances to monitor pollution levels at specific times.
3. *What are the observations generated by Sensor 14530?* This competency question enables users to view a series of time-based observations made by a sensor, facilitating analysis based on this temporal data.
4. *What is the average number of accesses recorded by Hospital Macchi during 2016 for digestive tract diseases?* This query provides information about hospital visits, which could have significant implications, such as links to data on water pollution for the same time frame.

As demonstrated by Table 5, in all cases, the WHOW-KG provides substantial additional value compared to other information sources. Extensive details are provided in the Supplementary information.

**Table 5:** Comparison of query results from SPARQL, Wikidata, and ChatGPT.

Query Description	SPARQL Result	Wikidata Result	ChatGPT Result
Number of water bodies in Lombardy (names and types)	840 results	96 results	7 results, only lakes
Concentration of reactive silicates for Lake Endine	38 results (observations)	Not applicable	No result
Observations generated by Sensor 14530	8928 results	Not applicable	No results
Average number of accesses by Hospital Macchi in 2016 for digestive diseases	1.05 (average value)	Page for the hospital exists, no details	No results

## Discussion

In this paper, we have introduced the Water Health Knowledge Graph (WHOW-KG) that links water quality observations with health parameters (e.g. infectious disease rates), thus implementing the well-known connection of water quality effects on people’s health. Our evaluations demonstrate the effectiveness of WHOW-KG in various aspects: for instance, the WHOW Ontology Network achieved a terminological coverage of 69.93% with GPT, significantly outperforming other ontologies. Additionally, the WHOW-KG provided substantially more detailed and relevant results for specific queries compared to other knowledge bases, such as Wikidata and ChatGPT, highlighting its richness and utility. The WHOW-KG is (i) distributed among different data providers, (ii) open to maximise re-use, (iii) multilingual in that labels and comments are provided in both Italian and English, when possible, and (iv) built according to FAIR principles, applied to both ontologies and linked open data. In this section, we will discuss our results in terms of impact, versioning and licensing, future plans and limitations.

### Impact

The UN Sustainable Development Goal (SDG) no. 6 on clean water and sanitation requires investing in adequate infrastructure, provide sanitation facilities, and encourage hygiene^95^. The importance of considering UN SDGs in the context of open data emerges from several contexts. Notable is the European Parliament resolution of 14 March 2019 on the Annual strategic report on the implementation and delivery of the SDGs (2018/2279(INI))^96^, where a precise call on the Commission is mentioned in order to add data related to the SDGs to the high-value datasets as defined in the directive on open data and public sector information, encouraging the Member States to publish all reports on the SDGs under a free license. The World Bank Group, in a blog post from as far back as 2015, explicitly highlights that “Open Data can help achieve the SDGs by providing critical information on natural resources, government operations, public services, and population demographics”^97^. To this end, the WHOW-KG embodies fine-grained thematic indicators that have been identified by data providers and co-creators of the WHOW project according to the three use cases and their legislation bases. Supported by the active participation of 77 individuals to the co-creation programme from different EU countries, we recorded evidence that the WHOW-KG is of utmost importance to the community encompassing decision makers, practitioners, and data providers in the area of water quality and sanitation.

### Limitations

While our study demonstrates the significant potential and effectiveness of the WHOW-KG, there are areas that require further attention and improvement. The WHOW-KG’s effectiveness is contingent on the availability and quality of underlying data sources. Secondly, while we have made strides in multilingual support, full coverage of all European languages remains an ongoing challenge. Additionally, the integration of LLMs in the evaluation, while promising, requires further validation to ensure consistent accuracy and performance. Finally, the current evaluation metrics, while comprehensive, could be expanded to include more qualitative assessments from end-users to better understand the practical utility and user satisfaction with the WHOW-KG.

### Future Plans

The WHOW-KG is continuously evolving with further datasets. The aim, in fact, is to provide a resource that can self-sustain and feed itself beyond the duration of the European WHOW project in which it was conceived. In this context, we are planning a number of activities to further increase the visibility of the knowledge graph and its use for any purpose of interest. Firstly, we are finalising the definition of SHACL shapes, starting from the OWL restrictions defined in the ontology network, to support the overall validation phase of the proposed methodology. Secondly, in order to open ourselves up to a wider audience of possible developers, part of our future work is to define REST APIs based on the semantics defined through the ontology network. Thirdly, in order to maximise the possibilities of re-use in a wider European context, we will exploit services such as eTranslation^98^ to provide additional languages for datasets and ontologies, making the knowledge graph understandable to possible stakeholders from different European countries. For what concerns the development of the ontology network, we are experimenting on AI-based approaches for knowledge engineering aspects tied to ontology design. To this regard, a preliminary experiment to generate ontological drafts from the WHOW competency questions methodologies has been made in Gangemi *et al*.^99^. Subsequently, an LLM-based methodology to draft ontologies basing on competency questions has been investigated in the Ontogenia methodology^100^. Our findings from these endeavors show that the use of LLMs for various aspects of Knowledge Graph generation and enrichment—from entity linking to ontology generation—is a promising area of study that has gathered increasing attention. This growing interest is evidenced by the special tracks dedicated to this topic at Semantic Web conferences: the Extended Semantic Web Conference (ESWC) 2024^101^, the International Semantic Web Conference (ISWC) 2024^102^ and the International Conference on Knowledge Engineering and Knowledge Management (EKAW) 2024^103^. As we continue to explore and refine the capabilities of LLMs, their potential to automate our use of Knowledge Graphs becomes ever more apparent.

## Supplementary information

Supplementary Information

## Data Availability

The WHOW-KG as the main outcome of the WHOW project provides a range of openly available resources accessible through various platforms. These resources include the project website, GitHub repositories for different aspects of the project, and SPARQL endpoints for querying data. The WHOW-KG is under version control on GitHub^52^. The ontology network, controlled vocabularies and linked dataset produced by ISPRA are released with a CC-BY 4.0^104^ license. Instead, the linked dataset produced by ARIA is released with a CC0^105^ license. Detailed descriptions of these resources and their links are provided below. **Project Resources:** 1. **Project Website** (https://whowproject.eu/): The main website for the WHOW Project, containing comprehensive information about the project and its objectives. 2. **Project Zenodo Community** (https://zenodo.org/communities/whow/): The community serves as a collector of records, publications, and deliverables related to the WHOW Project. 3. **Deliverables** (https://github.com/whow-project/deliverables): The project’s deliverables, including reports and documents, can be accessed on GitHub, although they are also available in our Zenodo Community as single resources for canonical citation. 4. **Hackathon** (https://github.com/whow-project/hackathon): The resources and outputs of the hackathons organised by the WHOW Project. 5. **ARIA SPARQL Endpoint** (https://lod.dati.lombardia.it/sparql): The ARIA SPARQL endpoint for querying linked data provided by the Lombardy Region. 6. **ISPRA SPARQL Endpoint** (https://dati.isprambiente.it/sparql): The ISPRA SPARQL endpoint for querying environmental data. 7. **The WHOW Knowledge Graph**, as presented in this work, is fully available on Zenodo for download and is composed of the following artifacts: • Ontology network is available on Zenodo^106^ under a CC-BY-4.0 license; • Controlled vocabularies are available on Zenodo^107^ under a CC-BY-4.0 license; • ISPRA Linked Open Data are available on Zenodo^108^ under a CC-BY-4.0 license; • ARIA Linked Open Data are available on Zenodo^109^ under a CC0 1.0 license. 8. **RML mappings files**: The mapping files represented as a RML (RDF Mapping Language)^47^ are available on Zenodo^49^ under a CC-BY-4.0 license. These resources collectively provide extensive information, datasets, and tools necessary to explore and reuse the data and outputs generated by the WHOW Project.

## Code availability

The WHOW Project coding resources are organised and described by their specific functions within the project. The main source code repository is publicly available on GitHub (https://github.com/whow-project).

The GitHub repositories also host the source code for the project’s ontologies and datasets, which are integral to the operation of the WHOW Project and can be accessed at https://github.com/whow-project/assets for ontologies and https://github.com/whow-project/datasets for datasets. The architecture and deliverables associated with the project are available at https://github.com/whow-project/architecture and https://github.com/whow-project/deliverables.

Hackathon resources, including the code used during these events, are provided at https://github.com/whow-project/hackathon. The code and other materials used in these hackathons are available for community use to encourage further development and integration into related projects.

For version-specific releases and additional code resources, the WHOW Project makes use of Zenodo to ensure long-term accessibility and reference. The Zenodo community can be found at https://zenodo.org/communities/whow.

Additionally, a list of the computational resources used to evaluate the WHOW-KG is available in Supplementary Information, along with other resources pertaining to the knowledge graph.
