# Supplementary material for: The Water Health Open Knowledge Graph
Source: Sci Data. 2025 Feb 15;12:274. doi: 10.1038/s41597-025-04537-4 (PMC11829977; doi:10.1038/s41597-025-04537-4)
Supplement: Supplementary file 1 — Supplementary Information [file 41597_2025_4537_MOESM1_ESM.pdf]

The Water Health Open Knowledge Graph
SUPPLEMENTARY MATERIAL

Anna Sofia Lippolis, Giorgia Lodi, Andrea Giovanni Nuzzolese

Table Of Contents

Supplementary Tables....... 2
WHOW-KG Resources....... 2
Supplementary Table 1. WHOW-KG Resources....... 2
WHOW-KG Evaluation Resources....... 2
Concept centrality evaluation – Degree Centrality Nodes....... 2
Supplementary Table 2. Degree Centrality Nodes....... 2
Concept centrality evaluation – Eigenvector Centrality Nodes....... 3
Supplementary Table 3. Eigenvector Centrality Nodes....... 3
Concept centrality evaluation – Betweenness Centrality Nodes....... 4
Supplementary Table 4. Betweenness Centrality Nodes....... 4
Terminological coverage evaluation....... 4
Supplementary Table 5. Keywords extracted by GPT-4....... 4
Supplementary Table 6. Keywords extracted by LLaMa....... 5
Supplementary Table 5. Keywords extracted by Claude....... 6
Supplementary Figures....... 7
Supplementary Figure 1: Top 10 Betweenness Centrality Nodes....... 7
Supplementary Figure 2: Top 10 Eigenvector Centrality Nodes....... 8
Supplementary Figure 3: Top 10 Degree Centrality Nodes....... 8

Supplementary Tables

WHOW-KG Resources

Supplementary Table 1. WHOW-KG Resources

Resource	URL
Project Website	https://whowproject.eu/
Project Zotero Community	https://zenodo.org/communities/whow/records?q=&l=list&p=1&s=10&sort=newest
Project GitHub Repository	https://github.com/whow-project
Ontologies	https://github.com/whow-project/assets
Datasets	https://github.com/whow-project/datasets
Architecture	https://github.com/whow-project/architecture
Deliverables	https://github.com/whow-project/deliverables
Hackathon	https://github.com/whow-project/hackathon
ARIA SPARQL Endpoint	https://lod.dati.lombardia.it/sparql
ISPRA SPARQL Endpoint	https://dati.isprambiente.it/sparql

WHOW-KG Evaluation Resources

Concept centrality evaluation – Degree Centrality Nodes

Supplementary Table 2. Degree Centrality Nodes.

Rank	URI	Score

1	https://w3id.org/whow/onto/hydrography/WaterBody	0.079
2	https://w3id.org/whow/onto/water-monitoring/DrinkingWaterObservation	0.068
3	https://w3id.org/whow/onto/health-monitoring/HealthcareIndicatorCalculation	0.068
4	https://w3id.org/whow/onto/water-monitoring/WaterObservablePropertyObject	0.056
5	https://w3id.org/whow/onto/water-monitoring/SurfaceOrGroundwaterObservation	0.056
6	https://w3id.org/whow/onto/water-monitoring/WaterMicrobiologicalParameterObservation	0.045
7	'https://w3id.org/whow/onto/hydrography/WaterFeature	0.045
8	https://w3id.org/whow/onto/water-monitoring/WaterObservation	0.045
9	https://w3id.org/whow/onto/water-monitoring/WaterHydromorphologicalParameterObservation	0.034
10	https://w3id.org/whow/onto/water-monitoring/RadioactivityObject	0.034

Concept centrality evaluation – Eigenvector Centrality Nodes

Supplementary Table 3. Eigenvector Centrality Nodes.

**Rank**	**URI**	**Score**
1	https://w3id.org/whow/onto/water-monitoring/DrinkingWaterObservation	0.49
2	'https://w3id.org/whow/onto/water-monitoring/SurfaceOrGroundwaterObservation	0.37

3	https://w3id.org/whow/onto/water-monitoring/WaterMicrobiologicalParameterObservation	0.36
4	'https://w3id.org/whow/onto/water-monitoring/WaterChemicalParameterObservation	0.312
5	https://w3id.org/whow/onto/water-monitoring/WaterIndicatorParameterObservation	0.312
6	https://w3id.org/whow/onto/water-monitoring/WaterObservation	0.27
7	'https://w3id.org/whow/onto/water-monitoring/WaterEmergingParametersObservation	0.22
8	https://w3id.org/whow/onto/water-monitoring/WaterHydromorphologicalParameterObservation	0.217
9	'https://w3id.org/whow/onto/water-monitoring/WaterPhysicoChemicalParameterObservation	0.217
10	https://w3id.org/whow/onto/water-monitoring/WaterBiologicalQualityParameterObservation	0.217

Concept centrality evaluation – Betweenness Centrality Nodes

Supplementary Table 4. Betweenness Centrality Nodes.

**Rank**	**URI**	**Score**
1	https://w3id.org/whow/onto/hydrography/WaterBody	0.0133
2	https://w3id.org/whow/onto/hydrography/WaterFeature	0.0109
3	https://w3id.org/whow/onto/water-monitoring/DrinkingWaterObservation	0.0086
4	https://w3id.org/whow/onto/water-monitoring/WaterObservation	0.00757
5	https://w3id.org/whow/onto/water-monitoring/SurfaceOrGroundwaterObservation	0.00705

6	https://w3id.org/whow/onto/health-monitoring/HealthcareIndicatorCalculation	0.00653
7	https://w3id.org/whow/onto/water-monitoring/WaterObservablePropertyObject	0.00496
8	https://w3id.org/whow/onto/water-monitoring/SamplingPoint	0.00287
9	https://w3id.org/whow/onto/health-monitoring/Population	0.00182
10	https://w3id.org/whow/onto/water-monitoring/WaterObservablePropertyObjectType	0.00156

Terminological coverage evaluation

Supplementary Table 5. Keywords extracted by GPT-4.

**Keyword**	**Score**
Water	1.301
Shall	1.257
Status	1.194
Groundwater	1.174
Conditions	1.171
Directive	1.170
States	1.153
Article	1.150
Bodies	1.148
Quality	1.145
European	1.138
Member	1.137
Surface	1.136
Type	1.121
Elements	1.107
Basin	1.105
River	1.101

Specific	1.100
Body	1.092
Measures	1.091

Supplementary Table 6. Keywords extracted by LLaMa.

**Keyword**	**Score**
water policy	0.95
european union	0.92
environmental protection	0.89
water quality	0.87
pollution prevention	0.85
integrated pollution prevention and control	0.83
directive 2000/60/ec	0.82
water framework directive	0.81
sustainable water management	0.79
groundwater protection	0.78
surface water	0.77
water abstraction	0.76
environmental objectives	0.75
water services	0.74
economic analysis	0.73
water pricing	0.72
cost recovery	0.71
public participation	0.70
river basin management	0.69
water management planning	0.68

Supplementary Table 7. Keywords extracted by Claude.

**Keyword**	**Score**

water	0.0625
Member States	0.0525
surface water	0.0475
groundwater	0.0425
river basin	0.0375
status	0.0325
quality	0.0325
monitoring	0.0325
ecological	0.0275
chemical	0.0275
programme	0.0225
objectives	0.0225
measures	0.0225
pollution	0.0225
directive	0.0225
environment	0.0175
protection	0.0175
management	0.0175
community	0.0175
substances	0.0175

Query evaluation on WHOW-KG, Wikidata, and ChatGPT

Supplementary Table 8: selected queries and results on the WHOW-KG

**CQ**	**SPARQL Query**	**WHOW-KG number of results**
How many water bodies are there in Lombardy, and what are their names and types?	PREFIX hydro: <https://w3id.org/whow/onto/hydrography/><br>PREFIX emf: <https://w3id.org/italia/env/onto/inspire-mf/>	840 results

	PREFIX rdfs:<br><http://www.w3.org/2000/01/rdf-schema#><br>SELECT ?mwb ?label ?tipo<br>WHERE{<br>?mwb a ?type ;<br>rdfs:label ?label .<br>?type rdfs:subClassOf* hydro:WaterBody;<br>rdfs:label ?tipo }	
What is the concentration of reactive silicates observed for the lake Endine?	PREFIX hydro:<br><https://w3id.org/whow/onto/hydrography/><br>PREFIX emf:<br><https://w3id.org/italia/env/onto/inspire-mf/><br>PREFIX top:<br><https://w3id.org/italia/env/onto/top/><br>PREFIX wm:<br><https://w3id.org/whow/onto/water-monitoring/><br>PREFIX data-lwb:<br><https://w3id.org/italia/lombardia/data/lake-water-body/><br>PREFIX data-op:<br><https://w3id.org/italia/lombardia/data/observable-properties/><br><br>SELECT ?obs ?time ?value ?unit<br>WHERE{<br>?obs a wm:WaterPhysicoChemicalParameterObservation ;	38 results

	emf:hasFeatureOfInterest data-lwb:endine ;<br><br>wm:hasWaterObservableProperty data-op:1344-09-8 ;<br><br>wm:hasResult/top:value ?value ;<br><br>wm:hasResult/top:hasUnitOfMeasure/top:name ?unit ;<br><br>emf:generationTime/top:time ?time .<br>}<br>ORDER BY ?time	
What are the observations generated by Sensor 14530?	PREFIX hydro: <https://w3id.org/whow/onto/hydrography/><br>PREFIX emf: <https://w3id.org/italia/env/onto/inspire-mf/><br>PREFIX top: <https://w3id.org/italia/env/onto/top/><br>PREFIX wh-mon: <https://w3id.org/whow/onto/weather-monitoring/><br>PREFIX sensor: <https://w3id.org/italia/lombardia/data/weather-sensor/><br>PREFIX data-op: <https://w3id.org/italia/lombardia/data/observable-properties/>	8928 results

	SELECT ?observation<br>?property ?feature ?time<br>?value<br>WHERE{<br>?observation<br>wh-mon:isObservationMadeBySensor sensor:14530 ;<br><br>wh-mon:hasObservedWeatherProperty ?property ;<br><br>wh-mon:hasFeatureOfInterest ?feature ;<br><br>wh-mon:hasResultTime<br>?time ;<br><br>emf:hasObservationValue<br>?value .<br>}	
What is the average number of accesses recorded by Hospital Macchi during 2016 for digestive tract diseases?	PREFIX ispra-top:<br><https://w3id.org/italia/env/onto/top/><br>PREFIX hm:<br><https://w3id.org/whow/onto/health-monitoring/><br>PREFIX indicator:<br><https://w3id.org/italia/lombardia/data/indicator/><br>PREFIX org:<br><https://w3id.org/italia/lombardia/data/inpatient-facility/><br>PREFIX disease:<br><https://w3id.org/italia/lombardia/data/disease-type/><br><br>SELECT ?calc ?value<br>WHERE{	1 result: “1.05”

	?calc a hm:HospitalCareIndicatorCalcul<br>ation ;<br><br>hm:doneForHCIndicator indicator:average-total-number-access ;<br><br>ispra-top:isIndicatorOf org:030901-01 ;<br><br>ispra-top:atTime/ispra-top:year "2016"^^xsd:gYear ;<br><br>hm:hasHealthcareIndicatorValue/ispra-top:value ?value ;<br><br>hm:ofClinicalCohort/hm:affectedBy disease:9<br>}	

Supplementary Table 9: selected queries and results on ChatGPT

CQ	Chat URL	Returned results
How many water bodies are there in Lombardy, and what are their names and types?	https://chatgpt.com/share/a80590ce-b3ec-4c6c-bd89-736cb4472397	7 results, only lakes
What is the concentration of reactive silicates observed for the lake Endine?	https://chatgpt.com/share/a80590ce-b3ec-4c6c-bd89-736cb4472397	No result
What are the observations generated by Sensor 14530?	https://chatgpt.com/share/a80590ce-b3ec-4c6c-bd89-736cb4472397	No result
What is the average number of accesses	https://chatgpt.com/share/a80590ce-b3ec-4c6c-bd89-736cb4472397	No result

recorded by Hospital Macchi during 2016 for digestive tract diseases?		

Supplementary Table 9: applicable query and results on Wikidata

**CQ**	**SPARQL query**	**Returned results**
How many water bodies are there in Lombardy, and what are their names and types?	Lakes:<br>SELECT ?waterBody ?waterBodyLabel<br>WHERE {<br>?waterBody wdt:P31 wd:Q23397;<br>wdt:P131* wd:Q1210. SERVICE wikibase:label { bd:serviceParam wikibase:language "[AUTO_LANGUAGE],en". }<br>}	47 results
	Rivers: SELECT ?waterBody ?waterBodyLabel<br>WHERE {<br>?waterBody wdt:P31 wd:Q4022;<br>wdt:P131* wd:Q1210.<br>SERVICE wikibase:label { bd:serviceParam wikibase:language "[AUTO_LANGUAGE],en". }<br>}	49 results

Supplementary Figures

Supplementary Figure 1: Top 10 Betweenness Centrality Nodes.

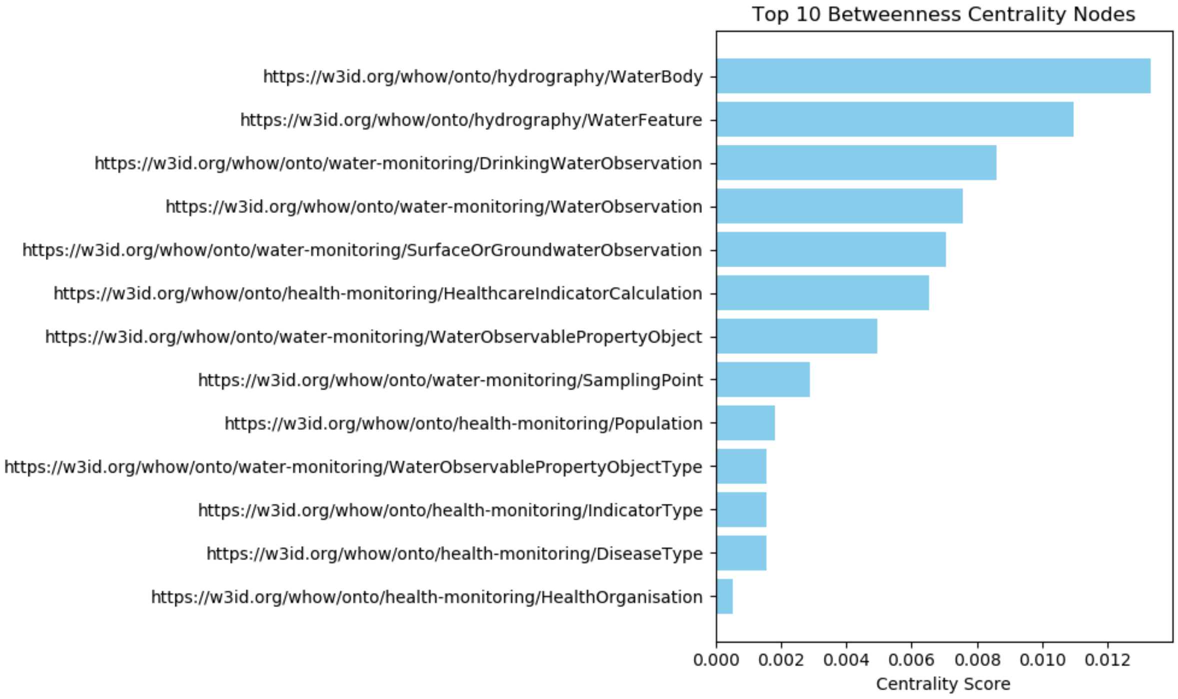

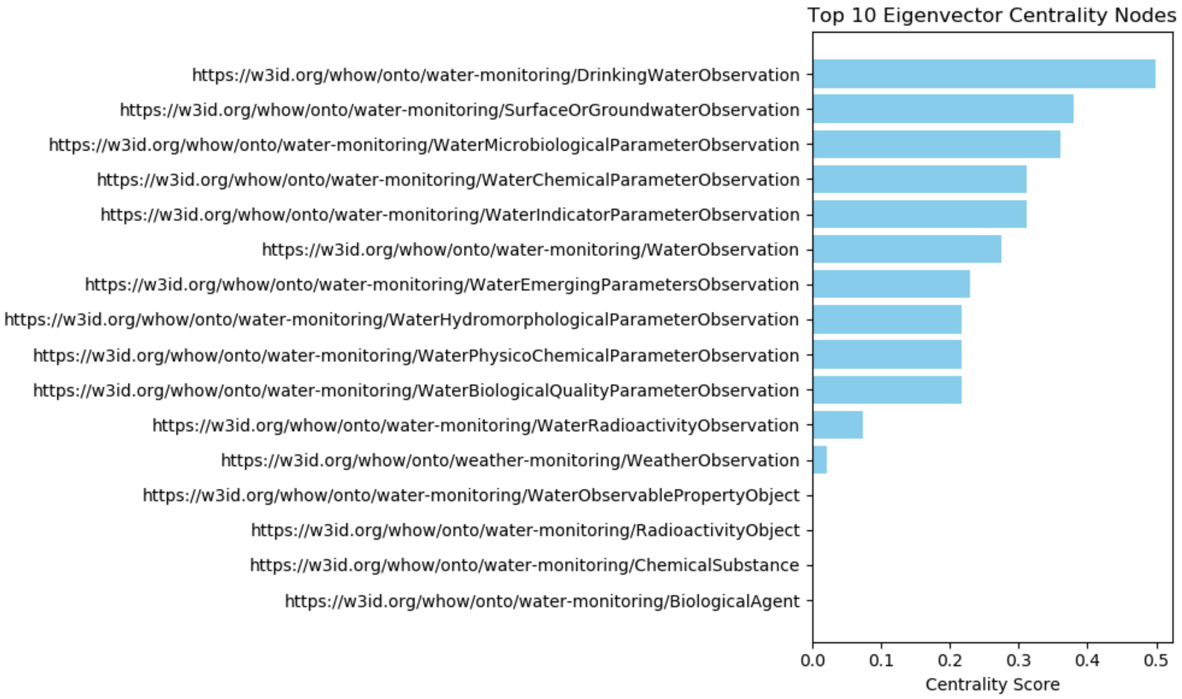

Supplementary Figure 2: Top 10 Eigenvector Centrality Nodes.

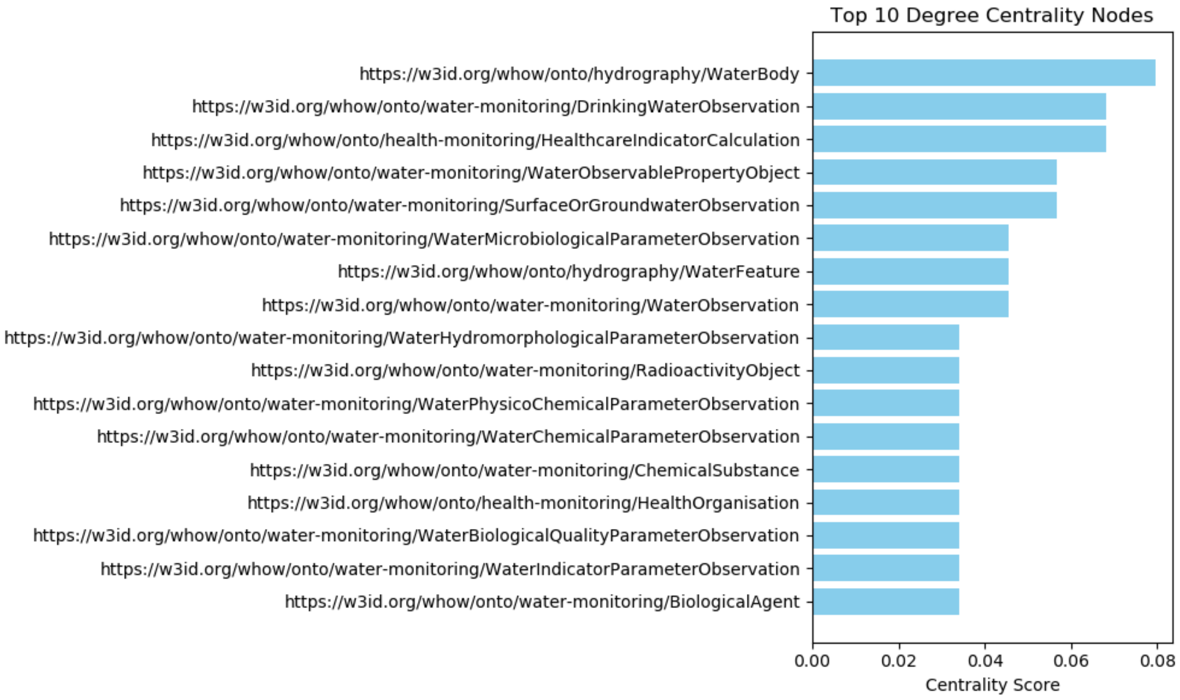

Supplementary Figure 3: Top 10 Degree Centrality Nodes.
